# RAMESES publication standards: realist syntheses

**DOI:** 10.1186/1741-7015-11-21

**Published:** 2013-01-29

**Authors:** Geoff Wong, Trish Greenhalgh, Gill Westhorp, Jeanette Buckingham, Ray Pawson

**Affiliations:** 1 Centre for Primary Care and Public Health, Queen Mary University of London, 58 Turner Street, London E1 2AB, UK; 2 Community Matters, P.O. Box 443, Mount Torrens, SA 5244, Australia; 3 John W. Scott Health Sciences Library, University of Alberta, Edmonton, AB T6G 2R7, Canada; 4 Department of Social Research Methodology, University of Leeds, Leeds LS2 9JT, UK

**Keywords:** realist synthesis, realist review, publication standards

## Abstract

**Background:**

There is growing interest in realist synthesis as an alternative systematic review method. This approach offers the potential to expand the knowledge base in policy-relevant areas - for example, by explaining the success, failure or mixed fortunes of complex interventions. No previous publication standards exist for reporting realist syntheses. This standard was developed as part of the RAMESES (Realist And MEta-narrative Evidence Syntheses: Evolving Standards) project. The project's aim is to produce preliminary publication standards for realist systematic reviews.

**Methods:**

We (a) collated and summarized existing literature on the principles of good practice in realist syntheses; (b) considered the extent to which these principles had been followed by published syntheses, thereby identifying how rigor may be lost and how existing methods could be improved; (c) used a three-round online Delphi method with an interdisciplinary panel of national and international experts in evidence synthesis, realist research, policy and/or publishing to produce and iteratively refine a draft set of methodological steps and publication standards; (d) provided real-time support to ongoing realist syntheses and the open-access RAMESES online discussion list so as to capture problems and questions as they arose; and (e) synthesized expert input, evidence syntheses and real-time problem analysis into a definitive set of standards.

**Results:**

We identified 35 published realist syntheses, provided real-time support to 9 on-going syntheses and captured questions raised in the RAMESES discussion list. Through analysis and discussion within the project team, we summarized the published literature and common questions and challenges into briefing materials for the Delphi panel, comprising 37 members. Within three rounds this panel had reached consensus on 19 key publication standards, with an overall response rate of 91%.

**Conclusion:**

This project used multiple sources to develop and draw together evidence and expertise in realist synthesis. For each item we have included an explanation for why it is important and guidance on how it might be reported. Realist synthesis is a relatively new method for evidence synthesis and as experience and methodological developments occur, we anticipate that these standards will evolve to reflect further methodological developments. We hope that these standards will act as a resource that will contribute to improving the reporting of realist syntheses.

To encourage dissemination of the RAMESES publication standards, this article is co-published in the Journal of Advanced Nursing and is freely accessible on Wiley Online Library (http://www.wileyonlinelibrary.com/journal/jan).

Please see related article http://www.biomedcentral.com/1741-7015/11/20 and http://www.biomedcentral.com/1741-7015/11/22

## Background

Academics and policymakers are increasingly interested in 'policy-friendly' approaches to evidence synthesis. Such approaches seek to illuminate issues and understand contextual influences on whether, why and how interventions might work [1,2]. A number of different approaches have been used to try to achieve this goal. At present there is lack of clarity on which methods are best suited for which questions or problems and this has been the subject of debate [3-6] and further research [7]. Realist synthesis is a theory-driven approach that is becoming increasingly popular.

### What is a realist synthesis?

In this section we briefly describe the realist synthesis method. The realist research question is often summarized as "What works for whom under what circumstances, how and why?" Realist inquiry is based on a realist philosophy of science and considers the interaction between context, mechanism and outcome. From a realist perspective, intervention X is not thought of as having effect size Y with confidence interval Z. Rather, intervention X (for example, a program introduced by policymakers who seek to create a particular outcome) alters context (for example, by making new resources available), which then triggers mechanism(s), which produce both intended and unintended outcomes. Intervention X may work well in one context but poorly or not at all in another context.

Realist inquiry seeks to unpack the context - mechanism - outcome relationship, thereby explaining examples of success, failure and various eventualities in between. Theoretical explanations of this kind are referred to as "middle-range theories" (that is, ones which "...involve abstraction... but [are] close enough to observed data to be incorporated in propositions that permit empirical testing" [8].

The basis of realist inquiry is a realist philosophy, whose key tenets are as follows:

1. There is a [social] reality that cannot be measured directly (because it is processed through our brains, language, culture and so on), but can be known indirectly.

Realism thus sits, broadly speaking, between positivism (‘there is a real world which we can apprehend directly through observation') and constructivism (‘given that all we can know has been interpreted through human senses and the human brain, we cannot know for sure what the nature of reality is').

2. Social programs (including complex interventions) may change the macro social context (for example, by introducing legislation). They may also change the resources or opportunities available to participants and, in that sense, change the meso- or micro-level context for those participants.

3. To understand the relationship between context and outcome, realism uses the concept of mechanisms, one definition of which is "...underlying entities, processes, or [social] structures which operate in particular contexts to generate outcomes of interest" [9].

In common with other theory-driven review methods, the realist approach offers the potential for insights that go beyond the narrowly experimental paradigm of the randomized controlled trial [10-12]. It can do so in relation to complex, complicated or simpler interventions (for example, even a simple intervention, such as a drug, is prescribed, dispensed and taken - or not - in a particular social, cultural and economic context).

"Realist synthesis" was first described by Ray Pawson in 2002 [13], updated in an ESRC (Economic and Social Research Council) commissioned monograph in 2004 [14], published as a book in 2006 [1] and summarizsed in a short methods paper in 2005 [15]. Since this paper is deliberately focused on publication standards, we strongly recommend that those unfamiliar with the realist approach consult these or other relevant methodological sources.

A realist synthesis (or realist review - these terms are synonymous) applies realist philosophy to the synthesis of findings from primary studies that have a bearing on a single research question or set of questions. Methodologically, reviewers may begin by eliciting from the literature the main ideas that went into the making of a class of interventions (the program theory). This program theory sets out how and why a class of intervention is thought to 'work' to generate the outcome(s) of interest. The pertinence and effectiveness of each constituent idea is then tested using relevant evidence (qualitative, quantitative, comparative, administrative and so on) from the primary literature on that class of programs. In this testing, the ideas within a program theory are re-cast and conceptualized in realist terms.

For each idea, reviewers seek out the contextual (C) influences that are hypothesized to have triggered the relevant mechanism(s) (M) to generate the outcome(s) (O) of interest. Synthesis consists of comparing 'how the programme was supposed to operate' to the 'empirical evidence on the actuality in different situations' - all along C-M-O lines. Analytic purchase comes from the ability to describe and understand the many contingencies that affect the likelihood of such interventions generating their intended outcomes. This in turn provides guidance about what policy makers or practitioners might put in place to change the context or provide resources in such a way as to most likely trigger the right mechanism(s) to produce the desired outcome.

### Why are publication standards needed?

Publication standards are common (and, increasingly, expected) - in health services research - see, for example, CONSORT for randomized controlled trials [16], AGREE for clinical guidelines [17], PRISMA for Cochrane-style systematic reviews [18] and SQUIRE for quality improvement studies [19]. For realist syntheses, publication standards are particularly important as this method is relatively new and concerns have been expressed about the rigor with which some realist reviews have been carried out and reported [20]. Publication standards are needed to ensure that users of reviews are provided with relevant and necessary information to enable them to assess the quality and rigor of a review.

In our experience, there is considerable confusion among researchers, journal editors, peer reviewers and funders about what counts as a high quality realist review and what, conversely, counts as a flawed review. Even though experts still differ on detailed conceptual methodological issues, the increasing popularity of this method prompted a study to develop baseline standards from which, we anticipate, further developments in theory and methodology of this approach will occur.

## Aim

The aim of this paper is to produce preliminary publication standards for realist syntheses.

## Methods

The methods we used to develop these reporting standards have already been published [20]. In brief, we purposively recruited an international group of experts to our online Delphi panel. Aiming to achieve maximum variety in the relevant sectors, disciplines and expert perspectives represented, we sought panel members working in realist research, evidence synthesis, publication, reviewer training and health policy. Prior to the start of our Delphi panel, with input from an expert informaticist (JB), we collated and summarized existing literature on the principles of good practice in realist synthesis, created a database of such published syntheses, and built relationships with teams who were undertaking ongoing syntheses. Through discussion within the project team, we considered the extent to which the principles had been followed by published and in-progress reviews, thereby identifying how rigor may be lost and how existing methods could be improved.

Our analysis of existing realist syntheses formed the basis of the briefing materials for the first round of the Delphi panel. In addition, we drew on our collective experience in training and supporting realist syntheses teams and an email discussion list on realist and meta-narrative methodology [21] to further inform the contents of our briefing document. Both the research team and panel members contributed draft items for the publication standards, and these were refined using the online Delphi process as previously described [20]. We ran the Delphi panels between September 2011 and March 2012.

### Description of panel and items

In all, we recruited 37 individuals from 27 organizations in 6 countries. These comprised: researchers in public or population health researchers (8); evidence synthesis (6); health services research (8); international development (2); education (2); and also research methodologists (6), publishing (1), nursing (2) and policy and decision making (2). In round 1, 22 Delphi panel members provided suggestions of items that should be included in the publication standards. In rounds 2 and 3 our panel members were asked to rate each potential item for relevance and clarity. The response rates across all items for rounds 2 and 3 were 93% and 89%, respectively. Consensus was reached within three rounds on both the content and wording of 19 items within the publication standards. Table 1 provides an overview of these items.

**Table 1:** List of items to be included when reporting a realist synthesis

TITLE		
1		In the title, identify the document as a realist synthesis or review

ABSTRACT		

2		While acknowledging publication requirements and house style, abstracts should ideally contain brief details of: the study's background, review question or objectives; search strategy; methods of selection, appraisal, analysis and synthesis of sources; main results; and implications for practice.

INTRODUCTION		

3	Rationale for review	Explain why the review is needed and what it is likely to contribute to existing understanding of the topic area.
4	Objectives and focus of review	State the objective(s) of the review and/or the review question(s). Define and provide a rationale for the focus of the review.

METHODS		

5	Changes in the review process	Any changes made to the review process that was initially planned should be briefly described and justified.
6	Rationale for using realist synthesis	Explain why realist synthesis was considered the most appropriate method to use.
7	Scoping the literature	Describe and justify the initial process of exploratory scoping of the literature.
8	Searching processes	While considering specific requirements of the journal or other publication outlet, state and provide a rationale for how the iterative searching was done. Provide details on all the sources accessed for information in the review. Where searching in electronic databases has taken place, the details should include, for example, name of database, search terms, dates of coverage and date last searched. If individuals familiar with the relevant literature and/or topic area were contacted, indicate how they were identified and selected.
9	Selection and appraisal of documents	Explain how judgements were made about including and excluding data from documents, and justify these.
10	Data extraction	Describe and explain which data or information were extracted from the included documents and justify this selection.
11	Analysis and synthesis processes	Describe the analysis and synthesis processes in detail. This section should include information on the constructs analyzed and describe the analytic process.

RESULTS		

12	Document flow diagram	Provide details on the number of documents assessed for eligibility and included in the review with reasons for exclusion at each stage as well as an indication of their source of origin (for example, from searching databases, reference lists and so on). You may consider using the example templates (which are likely to need modification to suit the data) that are provided.
13	Document characteristics	Provide information on the characteristics of the documents included in the review.
14	Main findings	Present the key findings with a specific focus on theory building and testing.

DISCUSSION		

15	Summary of findings	Summarize the main findings, taking into account the review's objective(s), research question(s), focus and intended audience(s).
16	Strengths, limitations and future research directions	Discuss both the strengths of the review and its limitations. These should include (but need not be restricted to) (a) consideration of all the steps in the review process and (b) comment on the overall strength of evidence supporting the explanatory insights which emerged. The limitations identified may point to areas where further work is needed.
17	Comparison with existing literature	Where applicable, compare and contrast the review's findings with the existing literature (for example, other reviews) on the same topic.
18	Conclusion and recommendations	List the main implications of the findings and place these in the context of other relevant literature. If appropriate, offer recommendations for policy and practice.
19	Funding	Provide details of funding source (if any) for the review, the role played by the funder (if any) and any conflicts of interests of the reviewers.

### Scope of the publication standards

These publication standards are intended to help researchers, authors, journal editors, and policy and decision makers to know and understand what should be reported in the write-up of a realist synthesis. They are not intended to provide detailed guidance on how to conduct such a synthesis; for this, we direct interested readers to summary articles [15,22] or various publications on methods [1,11,14,23]. This publication standard applies only to realist syntheses. A list of publication guidelines for other review methods can be found on the EQUATOR Network's website [24], but at present none of these relate specifically to realist syntheses. As part of the RAMESES project we are also developing quality standards and training materials for realist syntheses, which will be submitted as a separate publication. Publication standards for meta-narrative reviews (also covered in the RAMESES project) have been addressed in a separate article.

### How to use these publication standards

The layout of this document has drawn on previous methodological publications and, in particular, on the 'Explanations and Elaborations' document of the PRISMA statement [18]. Each item is followed by an example drawn from published reviews and a rationale for its inclusion. The purpose of the example text is to illustrate how an item might be reported in a write up. However, potentially relevant contextual information may have been omitted, so it may be necessary to consult the original paper from which the example text was drawn. The standards set out what might be expected for each item, but authors will still need to exercise judgement about how much information to include. The purpose of the details reported should be to ensure that the description and explanation provided is coherent and plausible, both against the guidance set out within an item and for the overall purpose of the realist synthesis.

While this publication standard is modeled on the PRISMA statement, the items within are not identical. This publication standard, developed to apply only to realist syntheses, has some overlap with the PRISMA statement. Items 1 to 3, 15, 16 and 19 in this statement broadly match the purpose of items 1 to 3, 24, 25 and 27 in the PRISMA statement. For items 4 to 14, while there is some overlap in purpose with some PRISMA statement items, different or additional reporting is needed due to the nature of realist syntheses. Other items (5, 12, 13, 15, 16, 19 and 23) in the PRIMSA statement have no equivalent in the RAMESES publication standards for realist reviews.

The order in which items are reported may vary. Realist syntheses are not 'linear' reviews. Some of the processes that are listed may legitimately take place in parallel or have to be revisited at a later date as a review progresses. As a general rule, if a recommended item is excluded from the write-up of a realist synthesis, a justification should be provided.

### The RAMESES publication standards for realist syntheses

#### Item 1: Title

In the title, identify the document as a realist synthesis or review.

### Example

"Human resource management interventions to improve health workers' performance in low and middle income countries: a realist review." [25]

### Explanation

Our background searching has shown that some realist reviews are not flagged as such in the title and may also be inconsistently indexed and, hence, are more difficult to locate during searching. The terms 'realist synthesis' and 'realist review' are both in widespread use. We asked our Delphi panel if they had a preferred term - 'realist synthesis' or 'review'. No consensus was reached by our Delphi panel on whether 'review' or 'synthesis' should be the preferred term, and there seemed no good reason to impose one or other term.

#### Item 2: Abstract

While acknowledging that requirements and house style may differ between journals, abstracts should ideally contain brief details of the study's background, review question or objectives; search strategy; methods of selection, appraisal, analysis and synthesis of sources; main results; and implications for practice.

### Example

#### "Background

Legislation is one of the most powerful weapons for improving population health and is often used by policy and decision makers. Little research exists to guide them as to whether legislation is feasible and/or will succeed. We aimed to produce a coherent and transferable evidence based framework of threats to legislative interventions to assist the decision making process and to test this through the 'case study' of legislation to ban smoking in cars carrying children.

#### Methods

We conceptualised legislative interventions as complex social interventions and so used the realist synthesis method to systematically review the literature for evidence. 99 articles were found through searches on five electronic databases (MEDLINE, HMIC, EMBASE, PsychINFO, Social Policy and Practice) and iterative purposive searching. Our initial searches sought any studies that contained information on smoking in vehicles carrying children. Throughout the review we continued where needed to search for additional studies of any type that would conceptually contribute to helping build and/or test our framework.

#### Results

Our framework identified a series of transferable threats to public health legislation. When applied to smoking bans in vehicles; problem misidentification, public support; opposition; and enforcement issues were particularly prominent threats. Our framework enabled us to understand and explain the nature of each threat and to infer the most likely outcome if such legislation were to be proposed in a jurisdiction where no such ban existed. Specifically, the micro-environment of a vehicle can contain highly hazardous levels of second hand smoke. Public support for such legislation is high amongst smokers and non-smokers and their underlying motivations were very similar - wanting to practice the Millian principle of protecting children from harm. Evidence indicated that the tobacco industry was not likely to oppose legislation and arguments that such a law would be 'unenforceable' were unfounded.

#### Conclusion

It is possible to develop a coherent and transferable evidence based framework of the ideas and assumptions behind the threats to legislative intervention that may assist policy and decision makers to analyse and judge if legislation is feasible and/or likely to succeed." [26]

### Explanation

Apart from the title, an abstract is the only source of information accessible to searchers unless the full paper is obtained. The information in it must allow reviewers and/or users to decide if the review is relevant to their needs.

### Introduction section

The following items should be reported in the introduction section.

#### Item 3: Rationale for review

Explain why the review is needed and what it is likely to contribute to existing understanding of the topic area.

### Example

"A number of reviews on the subject have tried to examine evidence to improve the operationalization of interventions by CHWs [community health workers], including for child health. Lehmann *et al*. (Reference x1) and Lewin *et al*. (Reference x1) have reviewed evidence on CHW interventions in LMIC [low-middle income countries] and Haines *et al*. (Reference x1) have particularly so for child health. Lewin *et al*. (Reference x1) found lay health workers to be effective in specific areas in child health, when compared to usual care. Haines *et al*. (Reference x1) highlight the contextual nature of CHW's performance. Both caution that CHW interventions are not the panacea for all that ails the health systems in LMIC and that large scale CHW programmes should be initiated with great caution. Both raise questions about the applicability of findings to different settings and about the conditions under which CHW interventions should be implemented." [27]

### Explanation

As with all research, a background section explaining what is already known and what the researchers considered to be the 'knowledge gaps' is a helpful orientation.

#### Item 4: Objectives and focus of review

State the objective(s) of the review and/or the review question(s). Define and provide a rationale for the focus of the review.

### Example

"The overriding question for the review was: Does moving from high-poverty neighborhoods to lower-poverty neighborhoods improve health? More specifically: What were the key health outcomes? Who experienced these outcomes? What appeared to be the mechanisms and associated context leading to the outcomes? As the review proceeded, it became clear that one of the only relatively consistent and statistically significant positive health outcomes was an improvement in mental health for adult women, children and adolescent girls. In this paper a review of mental health outcomes of MTO [Moving To Opportunity] is presented, along with some insights about the mechanisms and contexts through which the intervention appears to have impacted mental health." [28]

### Explanation

A realist research question contains some or all of the elements of 'What works, how, why, for whom, to what extent and in what circumstances, in what respect and over what duration?' and applies realist logic to address the question (see Item 11).

Because a realist synthesis may generate a large number of avenues that might be explored and explained, and because resources and timescale are invariably finite, the expectation is that the review must be 'contained' by progressively focusing both its breadth (how wide an area?) and depth (how much detail?). This important process may involve discussion and negotiation with, for example, content experts, funders and/or users. It is typical and legitimate for the synthesis' objectives, question and/or the breadth and depth of the review to evolve as the review progresses. How and why it evolved is usually worth reporting.

### Methods section

The following items should be reported in the methods section.

#### Item 5: Changes in the review process

Any changes made to the review that was initially planned should be briefly described and justified.

### Example

"As the review progressed we became aware of various data suitability limitations (see Discussion) and the emergence of two prominent demi-regularities prompted us to narrow our review focus to the two candidate theories discussed below." [29]

### Explanation

A realist synthesis can (and, in general, should) evolve over the course of the review. For example, changes to the research question or its scope are likely to have an impact on many of the synthesis' subsequent processes. However, this does not mean the synthesis can meander uncontained. An accessible summary of what was originally planned (for example, as described in an initial protocol) and how and why this differed from what was done should be provided as this may assist interpretation.

#### Item 6: Rationale for using realist synthesis

Explain why realist synthesis was considered the most appropriate method to use.

### Example

"Previous reviews sought to understand PR [participatory research] and provide practical recommendations (References x6) and to assess the value of PR to research goals, health status, and systems change (References x6). Nonetheless, the assessment of outcomes remains weak (Reference x4), partly because the methodologies used have generally failed to embrace the complexity of programs or address mechanisms of change (Reference x1). ...

To handle such complexity, we chose a realist approach (Reference x1) because it provides a rationale and tools for synthesizing complex, difficult-to-interpret evidence from community-based programs." [30]

### Explanation

Realist synthesis is a theory-driven method that is firmly rooted in a realist philosophy of science. It places particular emphasis on understanding causation (in this case, understanding how programs and policies generate outcomes through human decisions) and how causal mechanisms are shaped and constrained by social context. This makes it particularly suitable for reviews of certain topics and questions - for example, complex social programs that involve human decisions and actions. It also makes realist synthesis *less *suitable than other review methods for certain topics and questions - for example, those which seek primarily to determine the average effect size of a simpler intervention administered in a single or limited range of conditions. In our analysis of 37 published realist syntheses, the most common limitation was inadequate engagement with realist explanatory principles and the implications these have, first, for understanding programs and how they work, and second, for cumulating evidence and explanation.

Some realist syntheses published to date have deliberately adapted the method as first described by Pawson. Sometimes, adaptations may be entirely justifiable, but at other times they may indicate a poor grasp of realist methodology. To enable judgement to be made on adaptations, the description and rationale for adaptations should be provided. Such information will allow criticism, debate and counter criticism among review teams and users on the suitability of such adaptations, and may well facilitate methodological development.

#### Item 7: Scoping the literature

Describe and justify the initial process of exploratory scoping of the literature.

### Example

**Figure 1:**
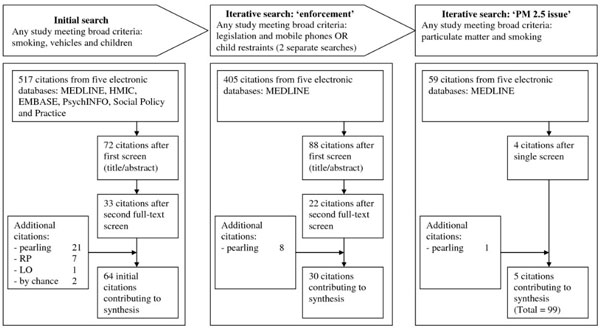
**Flow diagram illustrating search process and article disposition **[26].

"To develop our framework on the threats to the programme theory of public health legislation we started out by conducting a rapid review of broad areas of public health legislation (covering everything from gun amnesties to food labelling) trying to uncover what had been the sticking points in legislation and how (if at all) they had been circumvented. This outline review led to the construction of a provisional framework for reviewing the family of legislative interventions (as described in Figure 1). .... . Beginning with this framework and through discussions (and with reference to other interested stakeholders) we focused on a subset of themes that seemed most relevant in respect to the intervention in question. In our case, we deliberately sought input from the NICE officer seconded to our project." [26]

### Explanation

This step is used to build an understanding of the topic area. For example, this step may be used to identify provisional program theories, the names/titles of programs within scope and key authors in the area. Initial attempts to make sense of a topic area may involve informal 'browsing' of the literature and also consulting with experts and stakeholders.

#### Item 8: Searching process

While considering specific requirements of the journal or other publication outlet, state and provide a rationale for how the iterative searching was done. Provide details on all the sources accessed for information in the synthesis. For example, where electronic databases have been searched, details should include, for example, the name of the database, search terms, dates of coverage and date last searched. If individuals familiar with the relevant literature and/or topic area were contacted, indicate how they were identified and selected.

### Example

"...the literature search was iterative and ongoing throughout the project. An initial search was conducted of various academic databases, such as Academic Search Premier, Arts and Humanities Citation Index, Canadian Research Index, as well as through various search engines, such as Prowler, Novanet, Google and Google Scholar. Search terms included: Moving to Opportunity [MTO]; housing intervention; housing mobility; housing health effects; low-poverty neighborhood/community; high-poverty neighborhood/community; neighborhood/community health; poverty neighborhood/community; poverty community effects; poverty housing; poverty health; and housing health. A "snowball" approach was used in which one reference led to others. Other evaluations were revealed through correspondence with Dr. Jeffrey Kling, one of the principal MTO researchers." [28]

### Explanation

Searching should be guided by the objectives and focus of the synthesis, and revised iteratively in the light of emerging data. Data relevant to a realist synthesis may lie in a broad range of sources that may cross traditional disciplinary, program and sector boundaries. The search phase is thus likely to involve searching for different sorts of data, or studies from different domains, with which to test different aspects of any provisional theory.

Search methods using forward and backward citation tracking may be particularly valuable in finding the documents necessary to develop and then test provisional theories. Realist syntheses do not exclude sources solely on the basis of their study design; hence, 'methodological filters' (for example, to identify randomized controlled trials) may add little to the search and could potentially miss relevant papers.

Searching is likely to be iterative because, as the synthesis progresses, new or refined elements of theory may be required to explain particular findings, or to examine specific aspects of particular processes. As new elements of theory are included, searches for evidence to support, refute or refine those elements may be required. If undertaken, the process used for any such additional searches should be clearly documented. A single pre-defined search is unlikely to be sufficient and may suggest insufficient reflection on emerging findings.

Sufficient detail should be given to enable the reader to judge whether searching was likely to have located sources needed for theory building and/or testing.

#### Item 9: Selection and appraisal of documents

Explain how judgements were made about including and excluding data from documents, and justify these.

### Example

"Three tools were developed (for identification, selection, and appraisal) in March, June, and October 2009, respectively. Modifications were made during each stage after piloting. Each stage processed a different type of data: citations in identification; full-text papers in selection; and sets of publications in appraisal.

...The identification tool consisted of three questions. This step funnelled the number of citations from 7,167 to 594.

The librarian (JH) retrieved the 594 full-text papers, which were read by two independent reviewers, using a selection tool initially comprised of six questions in June 2009, with an additional two questions added in October 2009. ...

Two hundred articles remained from 594 after filtering them through the selection tool. Due to the complexity of the dataset, we decided at this stage to further limit the scope of our review to community-based settings, and to participatory interventions. Our rationale was that: PR in all forms (community-based PR, organizational PR, action research) was too diverse to be assessed within one review; the complexity of PR benefits from community-based research provided a manageable set of studies; intervention research demonstrated more complexity of outcomes than non-intervention research, and would be best suited for analysis using realist review methods; and the pool of studies needed to be reduced to a manageable size for an in depth realist synthesis (analysis). Adding two questions reduced the pool to 83 studies.....

Contact with principal investigators of all full-text papers retained after selection was undertaken because descriptions of programs, methods and findings of PR interventions were found to be commonly described across a number of publications pertaining to the same intervention. It was thus necessary to confirm that we had complete sets of papers in order to fairly appraise projects according to the realist review approach. ... For each study, we then sent our list of papers to the corresponding author or PI, and asked them to confirm that we had the complete set, or to send us additional documents. ... Only those sets of studies in which the contacted researcher responded to our request were retained for appraisal.

.... The appraisal tool consisted of three questions. An additional 11 sets were eliminated after screening with the tool below, which left a total of 23 sets, comprising 276 documents that were retained for synthesis. See Appendix 4: ... for a complete breakdown of the number of cases retained at each stage." [30]

### Explanation

Realist synthesis is not a technical process - that is, following a set protocol will not guarantee that a review will be robust. Rather, it requires a series of judgements about the relevance and robustness of particular data for the purposes of answering a specific question.

Within any document, there may be several pieces of data that serve different purposes, such as helping to build one theory, refining another theory and so on. Therefore, the selection (for inclusion or exclusion) and appraisal of the contribution of pieces of data within a document cannot be based on an overall assessment of study or document quality. An appraisal of the contribution of any section of data (within a document) should be made on two criteria:

*• Relevance *- whether it can contribute to theory building and/or testing; and

*• Rigor *- whether the method used to generate that particular piece of data is credible and trustworthy.

A wide range of documents can potentially contribute to a realist synthesis. For example, outcome and impact studies, qualitative interviews, ethnography, questionnaire surveys, mixed-method case studies, and close reading of policies, business plans, websites, project initiation documents and 'gray literature' write-ups of programs may all contribute in different ways of identifying and elucidating program theories. Because of this range and realist review's focus on relevance and rigor, it can initially be difficult to 'whittle down' the number of documents that are potentially eligible for inclusion in a review. This process can only occur as the data sources are analyzed in detail. Thus, in practice, the selection and appraisal stage may need to run in parallel with the analysis stage.

It is unlikely that authors will be able to provide an in-depth description of each decision involved, but the broad processes used to determine relevance and assess rigor (for example, using quality standards appropriate to particular kinds of research to appraise documents or sections of documents; discussion and/or debate within a review team of a document's findings; or consulting experts about technical aspects of methods or findings) should be described. While the description of the processes followed will not allow the reader to draw firm conclusions about judgements made, it will give an indication of the coherence, plausibility and appropriateness of the processes used to inform those judgements.

#### Item 10: Data extraction

Describe and explain which data or information were extracted from the included documents and justify this selection.

### Example

"In order to identify key elements of importance to the success or failure of an intervention in a certain context using a realist perspective, information was gathered on the intervention, the context and the actual "working of the intervention" or the mechanisms. As we intended to discuss the strength of the evidence and the usefulness of the application of realist principles to already published studies, we developed a process of data analysis that was comprehensive and as objective and transparent as possible. Therefore, a data analysis matrix was developed by the team of authors (see Annex 2). During the development of this matrix, the team extensively discussed and defined terms (such as context, mechanisms and outcome) and evaluation levels (such as process, output and outcome)." [31]

### Explanation

In a realist synthesis, data extraction assists analysis and synthesis. Reporting on what was extracted and why can add to the transparency of the synthesis process.

The extracted data may consist of descriptions (for example, of the detail of what was done in a program), findings (for example, cure rates, mortality) or explanations about how and why the program may have worked in particular contexts. Of particular interest to the realist reviewer are data that support the use of realist logic to answer the review's question(s) - for example, data on context, mechanisms and outcome configurations, demi-regularities, middle-range and/or program theories. Realist synthesis is used for a wide range of research questions, so it is impossible to be prescriptive about what data should be extracted. However, the link between the research question and the category of data extracted should be clear.

#### Item 11: Analysis and synthesis processes

Describe the analysis and synthesis processes in detail. This section should include information on the constructs analyzed and describe the analytic process.

### Example

"Data synthesis was undertaken either by RP and/or GW and synthesis results were regularly shared and discussed within the review team to ensure validity and consistency in the inferences made. Specifically (where relevant), we attempted to identify prominent recurrent patterns of contexts and outcomes (demi-regularities) in the data and then sought to explain these through the means (mechanisms) by which they occurred. For example, we noted that in our included articles self-reported public support for a ban on smoking in vehicles carrying children was often found to be high amongst smokers. During data synthesis we would then aim to provide an explanation of this demi-regularity through the identification of mechanism(s). As we delved further into our included articles and beyond (through our aforementioned purposive searching) for an explanation, data emerged that smokers harboured within them the wish to want to protect children from harm and also regret at having started smoking. We interpreted these as (realist) mechanisms and, for the former, were able to find substantive (middle-range) theory in the form of the Millean principle [Reference x1] to explain its interaction with context to influence outcomes. When additional studies were sought to enable programme theory testing, data handling processes .... were repeated." [26]

### Explanation

In a realist synthesis, the analysis and synthesis processes occur iteratively and may be sequential or in parallel. At the center of any realist analysis is the application of a realist philosophical 'lens' to data. A realist analysis of data specifically seeks to analyze data using realist concepts. Specifically, realism adheres to a generative explanation for causation - that is, an outcome (O) of interest was generated by relevant mechanism(s) (M) being triggered in context (C). Within or across the included documents, recurrent patterns of outcomes (or demi-regularities) and their associated mechanisms and contexts (CMO configurations) are likely to occur.

During synthesis the goal is to make sense of the analyzed data using theory, at one of two levels. First, theory (or theories) may be sought, developed and/or refined to explain how it is that a program (or part of a program) achieves its outcomes (that is, the mechanism(s) operating within a program) and the contexts in which those mechanisms do and do not fire. This provides a realist program theory. Second, theory (or theories) may be sought, developed and/or refined to explain, at a somewhat more general level, the pattern of contexts, mechanisms and outcomes. A full realist analysis addresses both these levels and attempts to make sense of the relationship between these two levels. Syntheses which address only one level may also be considered realist syntheses assuming that they apply and demonstrate application of a realist philosophy of science. The level(s) of analysis chosen will depend on the review's focus. The theories used may have been developed and/or refined from the data and/or be refinement of existing substantive theory.

The key analytic process in realist review involves iterative testing and refinement of theoretically based explanations using empirical findings in data sources. Reviewers may draw on any appropriate analytic techniques to undertake this testing. Explanation and justification for the choice of techniques should be provided.

Ideally a description should be provided on how all the individuals involved in the review have been involved in the analysis and synthesis processes, and how these evolved as the review took shape.

### Results section

The following items should be reported in the results section.

#### Item 12: Document flow diagram

Provide details on the number of documents assessed for eligibility and included in the review with reasons for exclusion at each stage, as well as an indication of their source of origin (for example, from searching databases, reference lists and so on). You may consider using the example provided (which is likely to need modification to suit the data) in Figure 1.

### Example

"See Figure 1: Flow diagram illustrating search process and article disposition." [26]

### Explanation

A flow diagram provides an accessible summary of the sequence of steps and gives an indication of the volume of data included and excluded at each step.

#### Item 13: Document characteristics

Provide information on the characteristics of the documents included in the synthesis.

### Example

"Additional File 1 summarises ..., the context, the intervention, the mechanisms triggered and the reported outcomes. Additional File 1 shows that in all the trials, more than one type of intervention was applied to improve CHWs [community health workers] performance. It also shows that the outcomes are reported not in terms of CHW performance, but rather in terms of the consequences of their performance on specific health outcomes." [27]

### Explanation

A clear summary of the characteristics of included sources can add to the transparency of the synthesis and some characteristics may help readers judge the coherence and plausibility of inferences. Examples of possibly relevant characteristics of documents that may be worth reporting include, where applicable: full citation, country of origin, study design, summary of key main findings, use made of document in the synthesis and relationship of documents to each other (for example, there may be more than one document reporting on an intervention). While considering specific requirements of any particular publication, reviewers may wish to tabulate key characteristics.

#### Item 14: Main findings

Present the key findings with a specific focus on theory building and testing.

### Example

"Using this theoretical concept, we hypothesized that equitable partnerships, with the stakeholders' participation throughout the project, succeed largely through synergy. Through the synthesis process using CMO configuring, we refined the theory by demonstrating that synergy is both an outcome and a context for partnership development - so that when synergy generated positive outcomes (e.g., enhanced trust or improved data collection), those outcomes generated new synergy. Expanding this logic, we demonstrated how partnership synergy created momentum over time, producing resilience in the face of obstacles as well as sustaining health-related goals, extending programs and infrastructure, and creating new and unexpected ideas and activities." [30]

### Explanation

The defining feature of a realist synthesis is the nature of the theory(ies) it offers. Such a theory explains why a social program/intervention generates particular outcomes in particular contexts, in terms of one or more mechanisms - that is how the program's infrastructure and resources trigger particular decisions or behaviors in human participants. Program theories are usually 'middle-range' - that is, specific enough to generate propositions that can be tested about aspects of the program but sufficiently abstract to be applicable to other programs. Mechanisms are contingent: they are causal processes that have a tendency to occur in a particular set of conditions, but which do not always occur (because the circumstances have to be right for any particular mechanism to operate, and because many mechanisms can operate concurrently, sometimes cancelling each other out).

The validity of a review which is described as 'realist' and which talks about program theories or mechanisms but which expresses these as simple and linear relationships between variables should be questioned.

The findings of a realist synthesis consist largely of inferences about the links between context, mechanism and outcome and the theory(ies) that seek to account for these links. It is important that where inferences are made these are clearly articulated. Where possible, especially for key findings, it is important to include an explanation to show how these inferences were arrived at.

Transparency of the synthesis process can be demonstrated, for example, by including such things as a detailed worked example, verbatim quotes from primary sources, and (if appropriate) an exploration of disconfirming data (that is, findings which appeared to refute the program theory but which, on closer analysis, could be explained by other contextual influences).

When presenting inferences about context-mechanism-outcome configurations, reviewers should be clear about what they have categorized as context, what as mechanism and what as outcome. In a realist synthesis a mechanism involves the interaction between particular inputs (or resources) and human reasoning, which produces a particular outcome (or not).

More than one piece of data might be needed to support an inference. It is sometimes appropriate to build the argument for an inference as an unfolding narrative in which successive data sources increase the strength of the inference [32]. Provide enough details about each data item to identify its source and enable readers to make judgements about its relevance and rigor.

### Discussion section

The following Items should be reported in the discussion section.

#### Item 15: Summary of findings

Summarize the main findings, taking into account the synthesis' objective(s), research question(s), focus and intended audience(s).

### Example

"This realist review of 249 primary studies has produced two key findings which are important, if somewhat unsurprising. First, Internet-based courses must engage their target group of learners to use the technology. This is likely to occur only if the technology is perceived as 'useful' (e.g.increases access to learning or saves time) and 'easy to use', though benefits in the former can outweigh challenges in the latter. Second, 'interactivity' is highly valued by learners. Learners wanted to be able to enter into a dialogue with the course tutor, fellow students and/or a virtual tutorial and obtain ongoing feedback on their understanding and performance." [29]

### Explanation

In order to place the findings in the context of the wider literature and any specific policy need, it is necessary to summarize briefly what has been found. This section should be succinct and balanced, explaining the relevance of one or more key theories that emerged from the analysis and highlighting the strength of evidence for the main inferences. This should be done with careful attention to the needs of the main users of the synthesis.

#### Item 16: Strengths, limitations and future research directions

Discuss both the strengths of the review and its limitations. These should include (but need not be restricted to) (a) consideration of all the steps in the synthesis process and (b) comment on the overall strength of evidence supporting the explanatory insights that emerged.

The limitations identified may point to areas where further work is needed.

### Example

"We explicitly chose to do a realist review of the RCTs [randomized controlled trials] to see what they could additionally yield. While the CHWs [community health workers] were an important component of the interventions being tested in the RCTs, none of the RCTs under review explicitly focused on performance of the CHW as an outcome. The RCTs under review offered a fair amount of information about the interventions, only some information about context - allowing us to formulate only generic hypotheses. ...

... Authors seldom described or discussed the mechanisms that explained their study outcomes. We realise that the RCT design, the exacting reporting requirements and word limits of journals, restrict authors from sharing all their operational experiences. In addition RCTs tend to report average effects and not differential effects of interventions, and less so of the context and rarely of the mechanisms triggered by their interactions. This makes the RCTs less useful for answering the questions regarding how interventions work. These generic hypotheses seem to be recurring in the literature, however they have not been explicitly tested across contexts." [27]

### Explanation

Realist synthesis may be constrained by time and resources, by the skill mix and collective experience of the research team, by the scope of the review's questions or objectives and/or by anticipated or unanticipated challenges in the data. These should be made explicit so that readers can interpret the findings in the light of them. A common challenge in realist synthesis is that in order to focus the synthesis, some material is omitted at each successive stage. Some aspects of the topic area, therefore, end up being reviewed in detail and rich explanatory insights produced for these. Other aspects are neglected (relatively or absolutely). It is thus inevitable that in generating illumination, the synthesis will also cast shadows. These should be highlighted in the discussion so as to indicate areas where other syntheses might focus.

Strengths and/or limitations associated with any modifications made to the synthesis process should also be reported and justified.

#### Item 17: Comparison with existing literature

Where applicable, compare and contrast the synthesis' findings with the existing literature (for example, other reviews) on the same topic.

### Example

"We were unable to find any comparable attempt at providing an evidence-based-policy framework such as ours. However, we acknowledge that some sections of our framework may be found in sources we have not uncovered and also as tacit knowledge within the heads of seasoned practitioners (e.g. advocates or legislators). We do however hope that our attempts to develop and test it on our one 'case study' will make a primordial tool that will be useful to policy and decisions makers less well versed in the arena of public health legislation." [26]

### Explanation

Comparing and contrasting the findings from a synthesis with the existing literature may help readers to put these into context. For example, this item might cover questions such as: How does this synthesis compare to other reviews (for example, were they theory-driven?); What does this synthesis add?; Which body of work in particular does it add to?; Has this synthesis reached the same or different conclusion to previous reviews?; and Has it answered a question previously identified as important in the field?

#### Item 18: Conclusion and recommendations

List the main implications of the findings and place these in the context of other relevant literature. If appropriate, offer recommendations for policy and practice.

### Example

"Our realist review was based on a housing intervention in the United States, but the results can potentially be applied to urban centers in other nations that implement housing interventions that involve moving families. When a family moves, the experience is likely to be different for each member of the household, and differences in mental health outcomes of moving may occur (Reference x1). All communities, rich or poor, and irrespective of geographic location, should be viewed as complex systems, and as composed of people with social relationships that influence the functioning and health of community members." [28]

### Explanation

A clear line of reasoning is needed to link the findings (Results section) with the implications (Discussion and/or Conclusion). If the synthesis is small and preliminary, or if the coherence and plausibility of evidence behind the inferences is weak or moderate, statements about implications for practice and policy should be appropriately guarded.

If recommendations are given, these should take into account the focus of the synthesis and needs of the intended audience and be presented appropriately. The explanations in realist analysis are highly dependent on contextual influences. It follows that recommendations must be contingent (for example, only under certain contexts will a particular mechanism be triggered to generate the desired outcome) rather than statements that X should or should not be done.

#### Item 19: Funding

Provide details of funding source (if any) for the synthesis, the role played by the funder (if any) and any conflicts of interests of the reviewers.

### Example

"We gratefully acknowledge a financial contribution from the Dutch Development Cooperation (DGIS)." [25]

### Explanation

The source of funding for a synthesis and/or personal conflicts of interests may influence the research question, methods, data analysis and conclusions. No review is a 'view from nowhere', and readers will be better able to interpret the review if they know why it was done and for which sponsor.

If a synthesis is published, the process for reporting funding and conflicts of interest as set out by the publication concerned should be followed.

## Discussion

We have developed these publication standards for realist synthesis (which we view as synonymous with realist review) by drawing together a range of sources - namely, existing published evidence, a Delphi panel and comment, discussion and feedback from a mailing list, training sessions and workshops. We hope these standards will lead to greater consistency and rigor of reporting and, thereby, make the outputs of realist synthesis more accessible, usable and helpful to different stakeholders.

This publication standard is not a detailed guide of how to undertake a realist synthesis. Other resources, both published (see Background) and in preparation, are better suited for this purpose. These standards have been developed as a guide to assist the quality of reporting of realist syntheses and the work of publishers, editors and reviewers. As part of the RAMESES project, we will be developing and disseminating both training materials and quality standards for realist synthesis [20].

Because realist synthesis is used for a broad range of topics and questions, and because it involves making judgements and inferences rather then checking against or following a technical checklist, it is impossible to be prescriptive about what exactly must be done in a review. The guiding principle is that transparency is important, as this will help readers to decide for themselves if the arguments for the judgements made were reasonable, both for the chosen topic and from a methodological perspective. We strongly encourage review authors to provide detail on what they have done and how - in particular with respect to the analytic processes used. These standards are intended to supplement rather than replace the exercise of judgement by editors, reviewers, readers and users of realist syntheses. We have tried to indicate in each item where judgement needs to be exercised.

The explanatory and theory-driven focus of realist syntheses means that detailed data may need to be reported in order to provide enough support for inferences and/or judgments made. While developing these publication standards, it became apparent that in some cases the word count limitations imposed by journals did not enable review teams to fully explain aspects of their synthesis - such as how judgments were made or inferences arrived at. Alternative ways of providing the necessary detail may need to be found, such as online appendices or additional files available from authors on request.

Previous efforts to develop publication standards have sometimes been criticized for being too 'ivory-tower' and failing to take account of real-world problems faced by reviewers. In an effort to redress this problem in the RAMESES project, we sought from the outset to engage not just senior academics but also junior and mid-career researchers, practitioners, policymakers and publishers in the development of the standards and to capture real-life challenges of ongoing realist syntheses as these emerged.

## Conclusions

We have developed these publication standards for realist syntheses by drawing on a range of sources. Our hope is that these standards will lead to greater consistency and rigor of reporting and make the outputs of realist syntheses more accessible, usable and helpful to different stakeholders. Realist synthesis is a relatively new approach to evidence synthesis and with increasing use and methodological development, changes are likely to be needed to any publication standards. We hope to continue capturing and improving these publication standards, through our email list [21] and wider links and discussions with researchers and those who commission, sponsor, publish and use realist syntheses.

## Abbreviations

ESRC: Economic and Social Research Council; RAMESES: Realist And MEta-narrative Evidence Syntheses: Evolving Standards).

## Competing interests

The authors declare that they have no competing interests. The views and opinions expressed therein are those of the authors and do not necessarily reflect those of the HS&DR program, NIHR, NHS or the Department of Health.

## Authors' contributions

GWo carried out the literature review. JB searched the literature for realist syntheses. GWo, TG, GWe and RP analyzed the findings from the review and produced the materials for the Delphi panel. They also analyzed the results of the Delphi panel. GWo, TG, GWe and RP conceived of the study and participated in its design. GWo coordinated the study and ran the Delphi panel. All authors read and approved the final manuscript.

## Pre-publication history

The pre-publication history for this paper can be accessed here:

http://www.biomedcentral.com/1741-7015/11/21/prepub
